# Argon plasma modified nanocomposite polyurethane scaffolds provide an alternative strategy for cartilage tissue engineering

**DOI:** 10.1186/s12951-019-0477-z

**Published:** 2019-04-06

**Authors:** Michelle Griffin, Deepak Kalaskar, Peter Butler

**Affiliations:** 1 Division of Surgery & Interventional Science, University College London (UCL), London, UK; 2 Plastic and Reconstructive Surgery Department, NHS Foundation Trust Hospital, Royal Free London, Pond Street, London, UK; 3 The Charles Wolfson Center for Reconstructive Surgery, Royal Free Hospital, London, UK; 4 UCL Institute of Orthopaedics and Musculoskeletal Science, Division of Surgery and Interventional Science, University College London, Stanmore, Middlesex HA7 4LP UK

**Keywords:** Nanocomposite, Argon modification, Polyurethane, Plasma surface modification

## Abstract

**Background:**

Children born with a small or absent ear undergo surgical reconstruction to create a suitable replacement using rib cartilage. To overcome the donor site morbidity and long-term pain of harvesting rib cartilage, synthetic materials can be a useful alternative. Medpor, is the currently used synthetic polyethylene material to replace missing facial cartilage but unfortunately it has high levels of surgical complications including infection and extrusion, making it an unsuitable replacement. New materials for facial cartilage reconstruction are required to improve the outcomes of surgical reconstruction. This study has developed a new nanomaterial with argon surface modification for auricular cartilage replacement to overcome the complications with Medpor.

**Results:**

Polyurethanes nanocomposites scaffolds (PU) were modified with argon plasma surface modification (Ar) and compared to Medpor in vitro and in vivo. Ar scaffolds allowed for greater protein adsorption than Medpor and PU after 48 h (p < 0.05). Cell viability and DNA assays demonstrated over 14-days greater human dermal fibroblast adhesion and cell growth on Ar than PU and Medpor nanocomposites scaffolds (p < 0.05). Gene expression using RT-qPCR of collagen-I, fibronectin, elastin, and laminin was upregulated on Ar scaffolds compared to Medpor and PU after 14-days (p < 0.05). Medpor, unmodified polyurethane and plasma modified polyurethane scaffolds were subcutaneously implanted in the dorsum of mice for 12 weeks to assess tissue integration and angiogenesis. Subcutaneous implantation of Ar scaffolds in mice dorsum, demonstrated significantly greater tissue integration by H&E and Massons trichrome staining, as well as angiogenesis by CD31 vessel immunohistochemistry staining over 12-weeks (p < 0.05).

**Conclusions:**

Argon modified polyurethane nanocomposite scaffolds support cell attachment and growth, tissue integration and angiogenesis and are a promising alternative for facial cartilage replacement. This study demonstrates polyurethane nanocomposite scaffolds with argon surface modification are a promising biomaterial for cartilage tissue engineering applications.

**Electronic supplementary material:**

The online version of this article (10.1186/s12951-019-0477-z) contains supplementary material, which is available to authorized users.

## Introduction

Surgical reconstruction of ear cartilage is required after trauma, infection, burns and congenital diseases [1–5]. Microtia, translated from the Greek, means little ear and is the medical word to describe a small or absent ear in newborn babies. The surgical reconstruction of the ear was first described by Tanzer et al. who used a six-stage procedure [6]. Since then Brent and Nagata modified the technique to a two-stage procedure, which has now become the most commonly used method of autologous ear reconstruction [7, 8]. In this procedure, cartilage is harvested from the ribs and carved into an auricular shape to place beneath the subcutaneous tissue. At approximately 6-months post the first procedure, the patient undergoes a second procedure to elevate the framework from the side of the head by placing further rib cartilage under the original carved structure. The main advantage of this technique is that rib cartilage is from the patient and thus is biocompatible but can be limited due to the amount of available rib cartilage. Despite being the gold standard technique, there are drawbacks to this autologous technique. Harvesting of rib cartilage has many surgical risks including pneumothorax, atelectasis, chest-wall deformities and it may cause scar and post-operative pain [3, 9]. With time the cartilage can also resorb and warp in shape, creating unreliable end results. Furthermore, the good clinical outcomes originally described by Brent and Tanzer are not always achieved due to the technical challenges of performing the surgery [10]. Due to autologous rib cartilage providing potential donor site morbidity and the technical challenge of the surgery, synthetic materials can also be used to reconstruct the ear cartilage [11]. Several prefabricated ‘off the shelf’ framework materials have been developed for ear reconstruction. The most commonly used material is Medpor, a high-density polyethylene (HDPE, Medpor, Pores Surgical Inc.). Medpor was developed in the 1970s and first reported for use in 1983 for reconstructive purposes [12–14]. However, reports of infection and extrusion with Medpor have prevented its widespread use [14]. This has prompted the need to develop alternative materials to improve the outcomes of synthetic auricular reconstruction.

Nanomaterials provide an alternative source of materials for cartilage replacement that have dimensions that mimic the native extracellular environment allowing the surrounding cells to adhere to the material and lay down their own extracellular matrix (ECM). However, finding the optimal nanomaterial to replace cartilage has yet to be identified. Polyurethanes pose a frequently used polymer choice for several biomedical applications [15]. Polyurethanes have a broad range of mechanical properties from rigid materials to soft elastomers [15]. Due to encouraging biocompatibility and hemocompatibility already reported, our group have been developing a nanocomposite polyurethane to mimic human auricular cartilage. Our nanocomposite material consists of a polyhedral oligomeric silsesquioxanes (POSS) modified polyurethane.

We have previously demonstrated that nanocomposite polyurethanes can be useful to replace auricular cartilage [16]. Polyurethanes with a pore size of 150–250 μm allowed for tissue ingrowth over 12 weeks in a rodent model [16]. However, one of the main problems with polyurethane biomaterials for regenerative purposes is that their surface is hydrophobic. Hydrophobicity or wettability of the material has a direct effect on protein absorption, its conformation, and thus eventually on cell attachment and consequently levels of tissue integration and angiogenesis [17]. Surface modification of biomaterials enables their surface to become more favourable for protein adsorption and consequently cell adhesion [18]. If cells adhere to a biomaterial surface then the implant is anchored to the surrounding tissue, preventing extrusion and infection of the biomaterial.

The surface of a biomaterial can be functionalised using two main techniques including physical and chemical modification to improve cell adhesion [19]. Physical modification can include modification of the surface topography to include pits or troughs to improve cell adhesion [20, 21]. However, this does not always provide the required molecules to induce appropriate signalling pathways for cell adhesion and differentiation [19]. Physical modification also includes the coating of biomaterial surfaces with ECM cell adhesive proteins including fibronectin, vitronectin, collagen or laminin [19]. The coating elicits cell directed new tissue formation by mediating biomolecular recognition. However, the correct protein, dose, orientation and half-life of the protein to achieve desired tissue responses are still under investigation [19]. Furthermore, the protein is susceptible to proteolytic degradation in the in vivo environment, limiting the potential clinical application of this approach [19]. Alternatively chemical modification includes the immobilization of various bioactive molecules on the surface. Covalent immobilization of RGD peptides or small protein fragments has shown to improve cell adhesion on different biomaterial surfaces [22, 23]. This approach has many advantages but is also associated with certain limitations [19]. Scaffold morphology can cause difficulties for chemical immobilisation, preventing optimal number and orientation of reactive sites for the molecules [19]. To overcome the limitations of these approaches would be to find a technique, which can modify the surface’s topography and induce a chemical functionalization.

Plasma surface modification (PSM) is one established technique, which creates a hydrophilic surface and can improve cell adhesion. PSM modifies the surface topography and immobilises chemical functional groups onto the surface [24–26]. Plasma is defined as a gas in the ionised state and was introduced by Langmuir in 1928 [27]. Applying a potential through a gas generates plasma modification. Radiofrequency plasma is the most widely used biomaterial surface treatment and involves passing an electric current through a gas at low or high pressure. Several studies have shown the beneficial effects of PSM on cell adhesion, proliferation and differentiation due to the surface topographical and chemical immobilization it creates [16, 25–28]. Hence, this study modified nanocomposite polyurethane implants with PSM to make it a suitable replacement for auricular reconstruction. We have shown previously that argon PSM is a superior surface modification technique compared to nitrogen and oxygen plasma treatment in improving cell adhesion, extracellular growth formation and tissue integration and vascularisation in vivo [28]. The surface’s physiochemical properties of the argon modified polyurethanes scaffold’s has been fully characterised demonstrating a hydrophilic and etched surface compared to unmodified polyurethane scaffolds [28].

To examine if PSM can be used to develop a suitable nanocomposites biomaterial for cartilage replacement, this study aimed to compare the modified polyurethane against Medpor. This study has conducted an in depth in vitro and in vivo investigation of PU modified with argon plasma (Ar), unmodified polyurethane (PU) and Medpor to evaluate differences in tissue integration, angiogenesis and immune response. This study provides evidence that nanocomposites polyurethane with argon surface modification holds promise provides an alternative scaffold for cartilage tissue engineering.

## Materials and methods

### Polyurethane manufacture and plasma surface modification (PSM)

In this paper, all in vitro and in vivo experiments were conducted on the three scaffolds; unmodified polyurethane (PU), argon modified polyurethane (Ar), and Medpor (0.85 mm thickness sheet, Porex Surgical, Newnan, GA, USA). PU scaffolds were synthesised and fabricated as described previously [16, 28–30]. In brief, polycarbonate polyol (2000 Mwt) and transcyclohexanechloroydrinisobutyl-silses-106 quioxane (Hybrid plastics Inc.) was mixed with POSS cages. To form the pre-polymer 4,4-methylenebis (phenyl 109 isocyanate) MDI was added to the solution. Then ethylenediamine and diethylamine was added slowly in DMAC to the mixture. The scaffolds were then fabricated using a previously described porogen leaching/solvent casting technique [16, 28–30]. In summary, the POSS-modified polycarbonate urea-urethane polymer solution was mixed with sodium chloride porogen (NaCl with pore size 150–250 μm) in a ratio of 1.1. The slurry was then mixed and degassed in a Thinky AER 250 mixer (Intertronics, Kidlington, UK). The mixture was then coated onto titanium moulds and heated for 4 h at 65 °C degrees. Following immersion in deionised water to dissolve the NaCl and DMAC for 24 h, the procedure was repeated until the final thickness of 0.85 mm was reached. Following the final layer, the scaffolds were further washed in deionised water for a further 7 days to ensure all the NaCl and DMAC was removed. Subsequently the PU scaffolds were modified with argon modification and referred to as Ar scaffolds. Unmodified PU scaffolds and referred to as PU. Argon modification of PU scaffolds was performed by exposing the scaffolds to 5 min using a radiofrequency plasma generator operating at 40 kHz with gas flow of 0.4 mbar at 100 W. Subsequently, for in vitro and in vivo experiments, 16 mm disks were cut from the polymer sheets.

### Assessment of HDF response in vitro

#### Cell culture and cell seeding

Human Dermal Fibroblast (HDFs) (European Collection of Cell Culture (ECACC) were maintained in Dulbecco’s Modified Eagle’s medium (DMEM) with 10% Foetal Bovine Serum (FBS) and 1% antibiotic solutions (Sigma, UK). For in vitro experiments, 16 mm scaffolds were placed into the 24-well plates, for 24 h in complete medium before cell seeding. Each scaffold was seeded with 1 × 10^4^ cells/cm^2^ unless stated. Every three days the medium was changed.

#### Assessment of cell morphology

The cell morphology of the HDFs was assessed after 24 h on PU, Ar and Medpor using F-actin staining as previously described [16]. In summary, 15,000 cells were seeded onto the scaffolds for assessment. After 24 h, the medium was removed from the scaffolds and washed with PBS several times. The cells were then fixed with 4% (w/v) paraformaldehyde in PBS at 37 °C for 10–15 min. After washing with 0.1% Tween-20 thrice and incubation with 0.1% TritonX-100 for 5 min to improve permeability the cells were stained with Rhodamine-conjugated phalloidin (ThermoFisher Scientific, UK) in the ratio 1:40. Following further washing, the cells were stained with DAPI (4′,6-diamidino-2-phenylindole, 1:500) to stain the nuclei. The scaffolds were then visualised to assess cell circularity and cell area (cell surface stained with actin) using ImageJ Software (n = 30 cells).

#### Assessment of cell adhesion and growth

The adhesion and growth of the HDFs onto the scaffolds was analysed using Fluorescence Hoechst DNA Quantification Kit (Sigma, UK) as described previously [16] after 1, 2, 4, 7, 10 and 14 days. In brief 200 μL of deionized deniosed water was added to the scaffolds at each time point followed by three freeze–thaw cycles to extract DNA from the scaffolds. A DNA standard curve was formulated by staining the DNA with bisBenzimide H 33258 solution, using the DNA standards provided. The fluorescence was measured with excitation and emission wavelengths of 360 nm and 460 nm using a Fluoroskan Ascent FL, (Thermo Labsystems, UK).

#### Gene expression

The gene expression of adhesion, extracellular matrix and angiogenesis was evaluated using RT-qPCR as previously described [30]. Firstly, RNA was extracted using Tri-Reagent (Life Technologies, UK). Then, Moloney murine leukemia virus reverse transcriptase was used to retro-transcribe the RNA (Promega, Madison, WI). Lastly, Real Time quantitative Polymerase Chain Reaction (RT-qPCR) was completed using with ABI Prism 7500 sequence detection system (Applied Biosystems) with QuantiTect SYBR Green PCR kit (Qiagen, Hilden, Germany). The housekeeping gene Glyceraldehyde 3-phosphate dehydrogenase (GAPDH) was used to normalise the data using the 2^−ΔΔCt^ method. Primer sequences and annealing temperatures for each set of primers was used as previously described [30].

#### Assessment of collagen production by HDFs

Collagen Type-I after 14 days was also examined using immunocytochemistry as previously described [31]. At 14 days, the medium was removed and the scaffolds were washed and fixed in 4% paraformaldehyde. Following incubation overnight, the scaffolds were washed with PBS. Subsequently, the scaffolds were permeabilized with (0.5% Triton x-100) and blocked with 0.5% Bovine Serum Albumin (BSA). Following incubation with primary antibody overnight at 4 °C 1:200 (abcam, uk ab34710), the scaffolds were washed thoroughly. After incubation with secondary antibody for 2 h (alexa fluor 488, 1:500) at room temperature, the cell nuclei were stained with Hoechst 33258 (2.5 μg/ml final concentration. Total collagen and hydroxyproline secretion was also examined after 7 and 14 days by the HDFs on the three scaffolds as previously described [32]. In brief, the extracellular total collagen production by cells was analysed using the PicroSirius Red (PSR) method as originally described (n = 6) [32]. Medium from the scaffolds was taken after to measure hydroxyproline content using a QuickZyme hydroxyproline assay kit (Biosciences) (n = 6) as described by previously [32].

#### Assessment of VEGF secretion by ELIZA using HDFs

The secretion of VEGF was examined from the HDFs after 4, 7, 10 and 14 days using ELISA analysis as previously described (ELISA, quantikine, R&D system, Abingdon, UK) [32]. In brief, cell culture supernatants at the required time points was centrifuged at 200*g* for 10 min and then stored at − 20 °C. The supernatants were then placed in ELISA 96-well plates coated with monoclonal antibody specific for VEGF. Following incubation for 2 h at room temperature, the enzyme linked anti-VEGF polyclonal antibody was added and incubated for a further 20 min. The colour was stopped using stop solution and the colour intensity measured with a colorimetric plate reader (Anthos 2020 microplate reader, Biochrome Ltd, UK) set at 450 nm and 540 nm wavelength.

### Assessment of immune response in vitro

Macrophage responses to the PU scaffolds were investigated by transformed U937 macrophage proliferation, cell morphology, and cytokines release assessments as previously described [32].

#### Macrophage cell culture

Macrophage cell culture was performed as described in Nayyer et al. [32]. In brief, the human promonocytic cell line U937, isolated from a histiocytic lymphoma (ECACC, UK, Number 85011440), was cultured in RPMI-1640 supplemented with 10% Foetal Bovine Serum (FBS), 5% l-glutamine (2 mM) and 1% antibiotic (50 μg/ml streptomycin, 50 U/ml penicillin) solutions (all from Sigma, UK) and incubated at 37 °C under a humidified atmosphere of 5% CO2. The nonadherent undifferentiated U937 was washed (× 3) every 3 to 4 days in Hank’s balance solution. Each 16 mm polymer disc in a 24 well plate was seeded with U937 cells at a density of 1 × 10^5^ cells/cm^2^ in 1 ml of cell culture medium supplemented with 0.5 nM phorbol-12- myristate 13-acetate (PMA) (Sigma, UK). Under these conditions, U937 monocytes will adhere to surfaces and differentiate into macrophage-like cells. After 24 h the wells were aspirated to remove any unattached and non-differentiated monocytes, and replaced with fresh medium. Lipopolysaccharide (LPS), a Gram-negative bacterium, (Sigma, UK) stimulated transformed macrophages on the polymer surfaces at a concentration of 1 μg/ml as a positive control.

#### Macrophage adhesion and growth

The adhesion and growth of the macrophages was assessed after 1, 4, 7 and 10 days on the materials using DNA assay as described above.

#### Macrophage cytokine release

Cytokines release including TNF-α, IL-10 and IL-β quantification using a sandwich ELISA assay (Quantikine, R&D System, Abingdon, UK) and performed in response to the different scaffolds after 1, 4, 7, and 10 days as previously described [32].

### In vivo analysis

Subcutaneous implantation of PU, Ar, and Medpor scaffolds was examined in a subcutaneous mouse implantation model for 6 and 12 weeks as described previously [16]. Of each scaffolds, two 4 mm discs were subcutaneously implanted in the dorsum of one 4-month-old BALB/c mice (Charles River Laboratories, UK) (*n* = 6). The disc was implanted via a small incision and closed with interrupted 5,0 monocryl. All experiments were approved by the local governmental animal care committee and performed according to animal welfare UK legislation. At 6 and 12 weeks, using CO_2_ asphyxiation the animal were scarified and the scaffolds excised in fixed in 4% paraformaldehyde. The scaffolds were excised with approximately 0.5 cm surrounding circular dermis. Fixed scaffolds were then paraffin embedded and cut into 3 μm sections for histological analysis. H&E staining, Masson Trichrome staining was conducted according to standard procedures to assess tissue integration and CD31 immunohistochemistry staining for endothelial cells detection as per a previous study [16].

### Statistical analysis

The comparisons between the three scaffolds were analysed statistically using one-way analysis of variance (ANOVA) with Tukey HSD post hoc analysis (Prism6 Software). In addition, the average and standard deviation (SD) was calculated. Significance was described as *p *< 0.05. Graphpad was used for graphically representing data.

## Results

### Protein adsorption

**Fig. 1:**
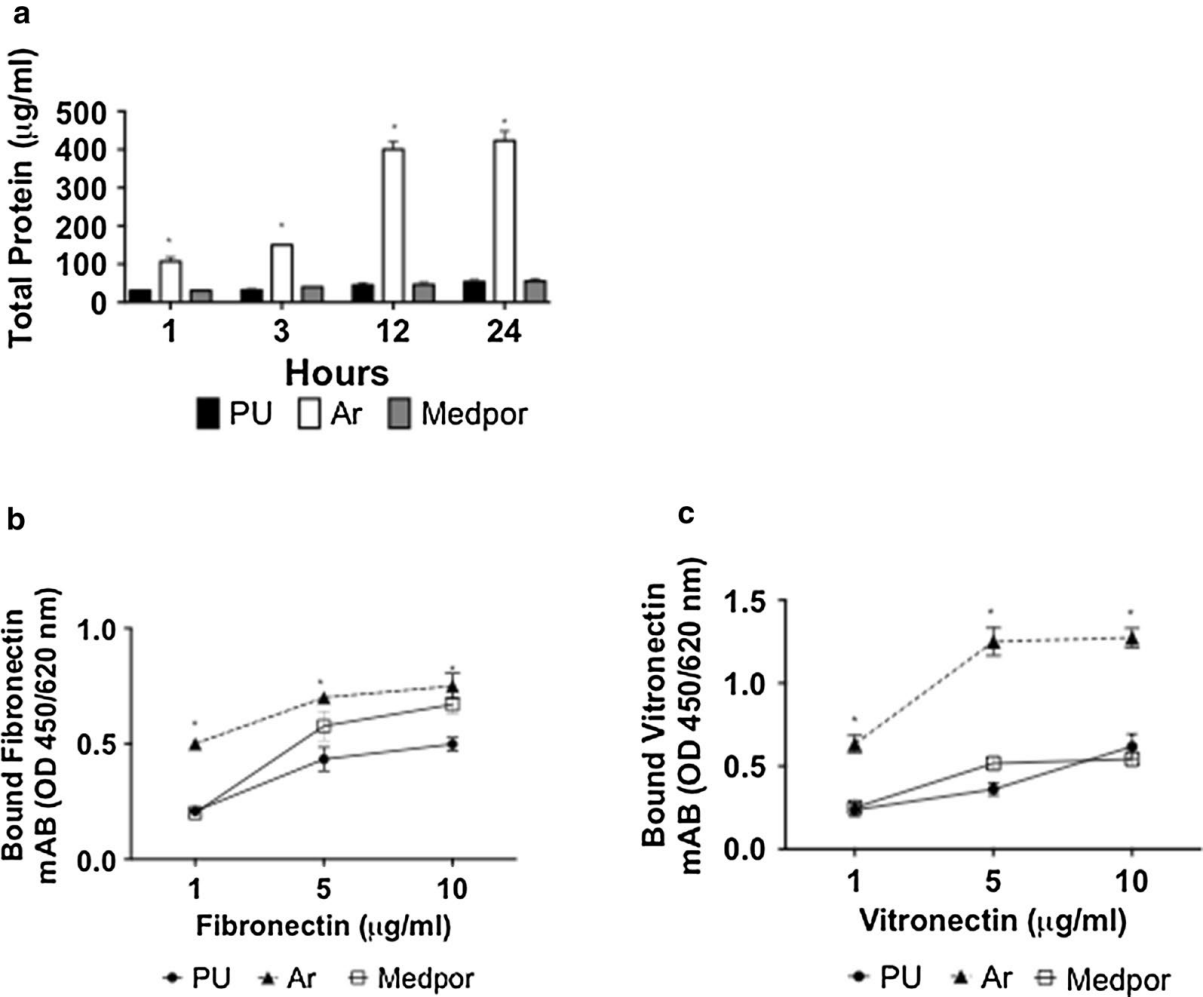
Protein adsorption studies on the polyurethane (PU), argon (Ar) and Medpor scaffolds. **a** Total protein adsorption on the Ar, Medpor, and PU scaffolds. Ar showed significantly greater protein adsorption compared to untreated PU and Medpor scaffolds (*p < 0.05). **b** Fibronectin and **c** vitronectin adsorption on the Ar, Medpor, and PU scaffolds. Ar scaffolds showed greater fibronectin adsorption than Medpor and PU scaffolds. Ar showed significantly higher vitronectin adsorption than Medpor and PU scaffolds. PU; Unmodified Polyurethane

As cells adhere via proteins to implant surface, the protein adsorption on the three surfaces was investigated. BCA assay showed the total protein adsorption onto the scaffolds was significantly the highest on Ar scaffolds after 24 h compared to Medpor and PU (Fig. 1a) (p < 0.05). PU showed similar BCA protein adsorption compared to Medpor over 24 h. Monoclonal antibodies were used to determine if fibronectin and vitronectin was adsorbed in the correct conformation on the different material surfaces (Fig. 1b, c). Vitronectin and fibronectin showed different levels of conformation on the scaffolds. Vitronectin was greatest on Ar surfaces and the lowest on Medpor scaffolds. The fibronectin adsorption was also significantly greatest on the Ar scaffolds compared to Medpor and POS-PCU scaffolds (p < 0.05).

### HDF in vitro assessment

#### HDF adhesion and growth

**Fig. 2:**
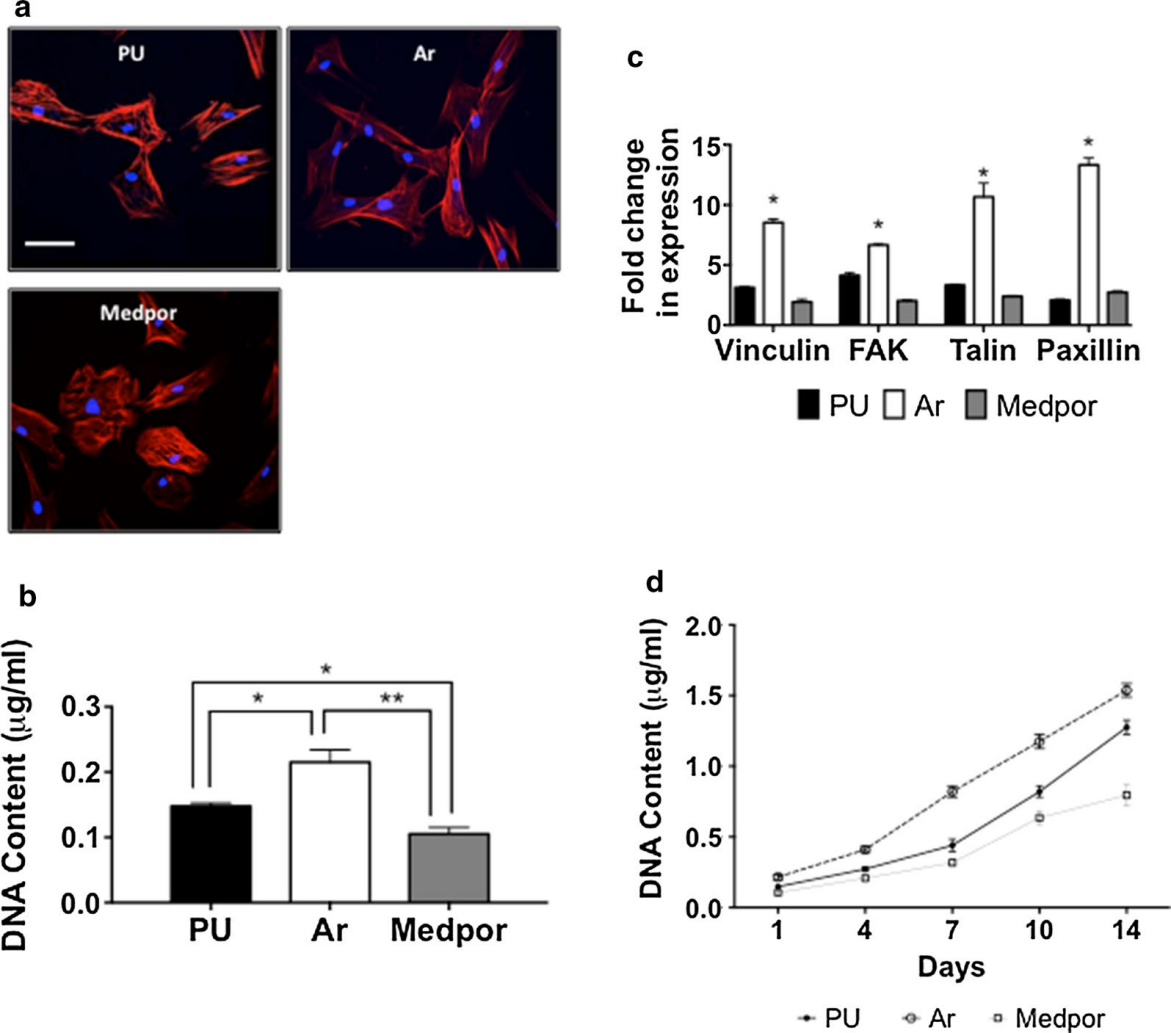
Human dermal fibroblast (HDF) adhesion in vitro studies on polyurethane (PU), argon (Ar) and Medpor scaffolds. **a** HDF morphology after 24 h using F-actin staining on the PU and Medpor scaffolds. Scale bar refers to 30 μm. **b** Human Dermal Fibroblast (HDF) adhesion after 24 h on PU and Medpor scaffolds. P values * < 0.05 ** < 0.01. **c** RT-qPCR analysis of adhesion related expression markers after 24 h on PU and Medpor scaffolds. Ar showed significantly greater expression of adhesion markers than Medpor and PU (PU vs Ar p < 0.05, Medpor vs Ar *p < 0.01). Fold changes represents difference compared to housekeeping gene GAPDH of HDFs cell growing on tissue culture plastic. **d** HDF DNA content over 14 days on PU and Medpor scaffolds. Ar showed greater DNA content at 1, 4, 7, 10 and 14 days compared to PU and Medpor. PU; Unmodified polyurethane

The morphology of the HDFs found showed a variety of morphologies on all surfaces. The HDF adopted a stretched morphology on Ar, Medpor, and PU scaffolds and were able to spread on the surfaces (Fig. 2a). The adhesion of the HDF was varied among the three surfaces after 24 h. DNA assay showed HDF adhesion was significantly higher on Ar than Medpor and PU scaffolds (p < 0.05) (Fig. 2b). Adhesion related genes including paxillin, talin, and vinculin were observed to be upregulated on the Ar scaffolds compared to the Medpor and PU scaffolds (p < 0.01) after 24 h (Fig. 2c). HDF cell growth was greatest over 14 days on Ar scaffolds compared to Medpor and PU scaffolds using DNA assay (p < 0.05) (Fig. 2d). HDF on PU scaffolds showed significantly higher levels of DNA content from day 7 compared to HDFs on Medpor scaffolds (p < 0.05).

#### HDF angiogenesis

**Fig. 3:**
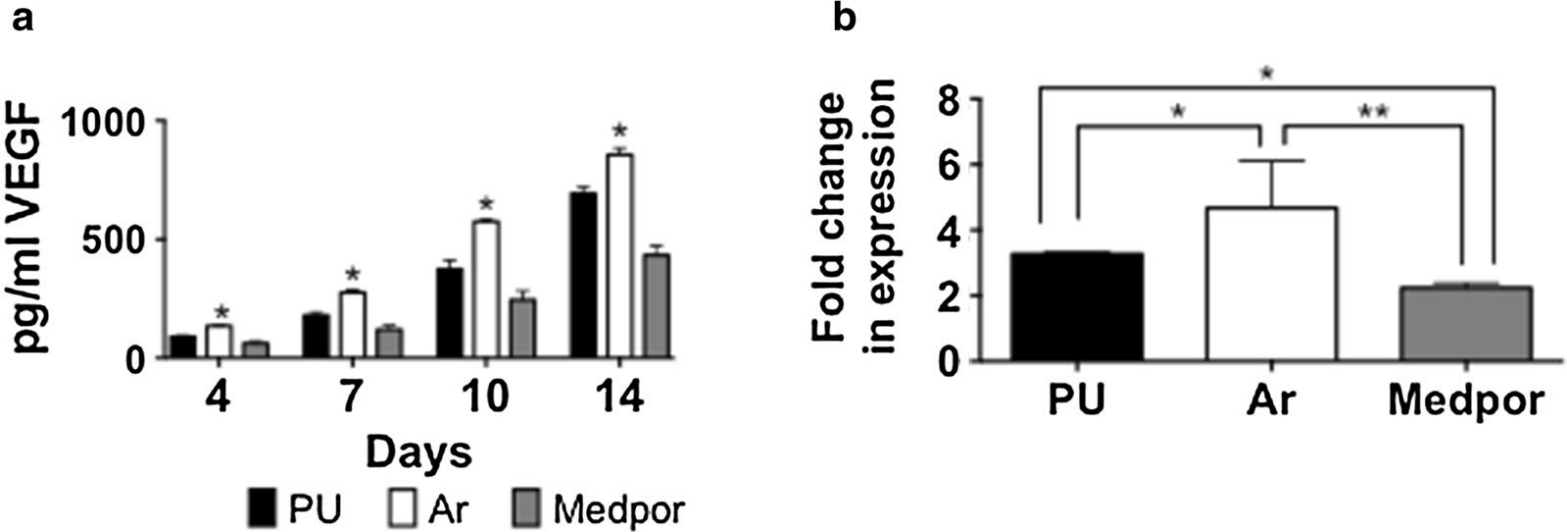
Assessment of angiogenesis on polyurethane (PU), argon (Ar) and Medpor scaffolds. **a** Vascular Endothelial Growth Factor (VEGF) secretions by the human dermal fibroblast (HDFs) on PU and Medpor after 4, 7, 10 and 14 days. **b** RT-qPCR of Vascular Endothelial Growth Factor (VEGF) on PU and Medpor scaffolds after 14 days. Fold changes represents difference compared to housekeeping gene GAPDH of HDFs cell growing on tissue culture plastic. P values * < 0.05 ** < 0.01. PU; Unmodified polyurethane

The secretion of VEGF protein by from the HDFs at 7 and 14 days, was the greatest on the Ar scaffolds compared to Medpor and PU (Fig. 3a). Medpor showed the lowest secretion of angiogenic protein VEGF from 4 to 14 days (p < 0.05). The mRNA expression of VEGF was also examined after 14 days to assess angiogenesis. The HDFs on Ar scaffolds showed the greatest mRNA expression of VEGF (p < 0.05) (Fig. 3b). The HDFs on PU showed a greater upregulation of mRNA expression of VEGF compared to HDF on Medpor (p < 0.05).

#### HDF extracellular matrix formation

**Fig. 4:**
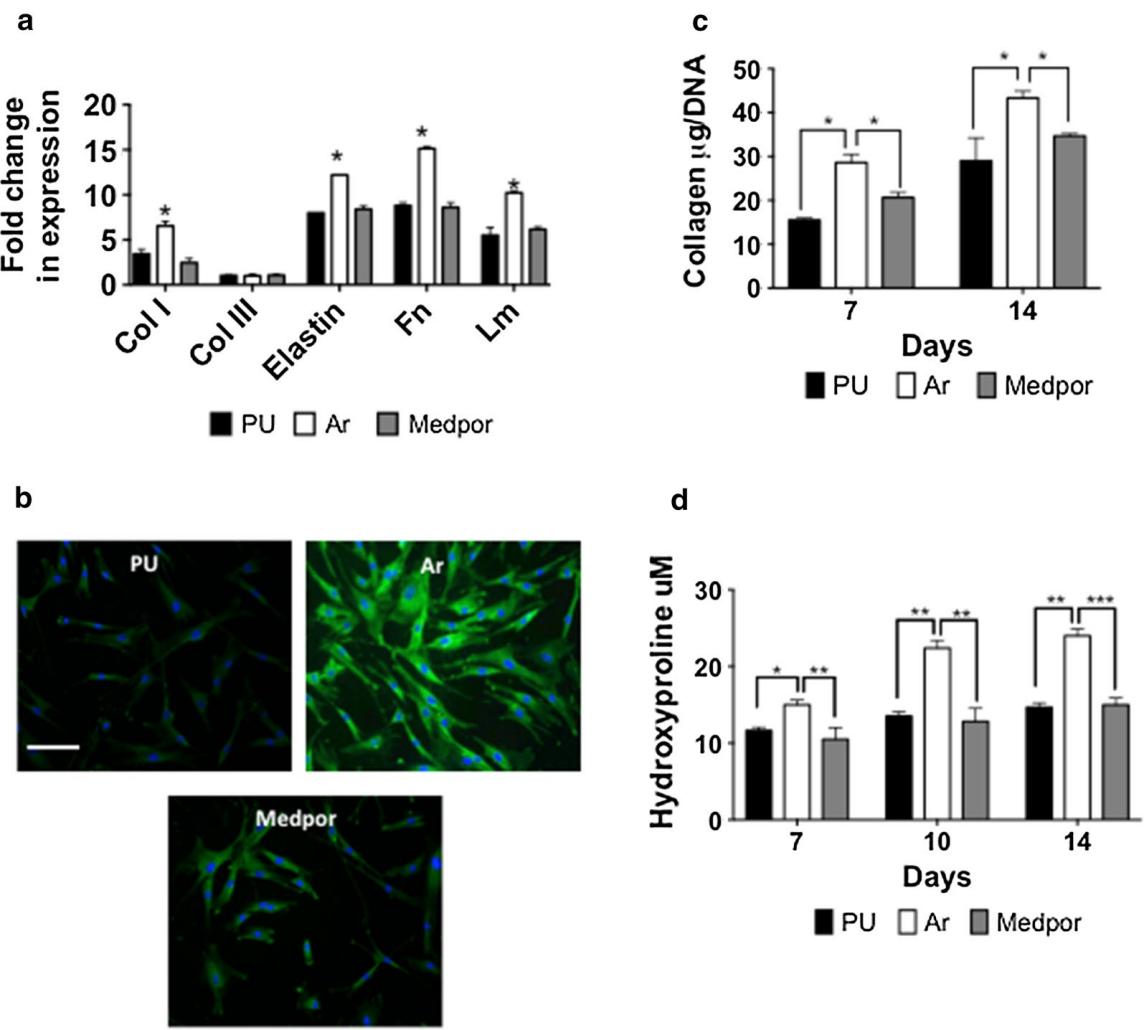
Assessment of Extracellular Matrix (ECM) formation by the human dermal fibroblasts (HDFs) after 14 days on polyurethane (PU), argon (Ar) and Medpor scaffolds. **a** RT-qPCR Analysis of ECM formation on all scaffolds. Ar showed a significantly greater expression of Collagen I (Col I), Collagen III (Col III), elastin, fibronectin (Fn) and laminin (Lm) after 14 days than Medpor and PU (*p < 0.05). Fold change represents difference to housekeeping gene GAPDH of HDFs growing on tissue culture plastic. **b** Collagen Type I expression by immunocytochemistry of the HDFs after 14 days on all scaffolds. Green; Collagen Type I, Blue: Nuclei staining using DAPI. Scale bar refers to 50 μm. **c** Total collagen secretion by the HDFs on all scaffolds. **d** Hydroxyproline secretion by the HDFs on all scaffolds. P values * < 0.05 ** < 0.01 ***p < 0.001. PU; Unmodified polyurethane

The expression of genes associated with formation of the extracellular matrix was examined including collagen I (col-I), collagen (col-III), fibronectin (Fn), elastin (elastin), and laminin (Lm) (Fig. 4a). The HDFs on Ar scaffolds showed increased mRNA expression at 7 and 14 days compared to HDF on PU and Medpor scaffolds. Staining of collagen type I confirmed the increased protein expression at 14 days by the HDF on Ar scaffolds (Fig. 4b). Total collagen secretion (Fig. 4c) and hydroxyproline secretion (Fig. 4d) by the HDFs was also elevated on the Ar scaffolds compared to Medpor and PU scaffolds (p < 0.05).

### Assessment of immune response in vitro

**Fig. 5:**
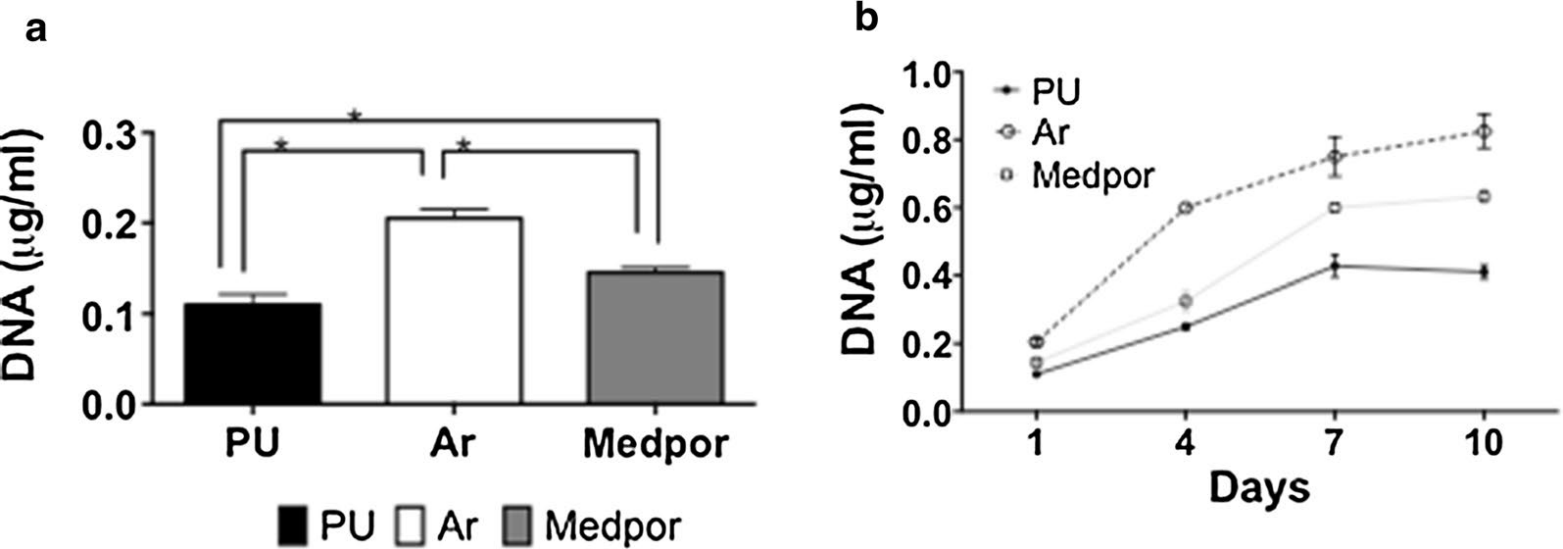
Human macrophage in vitro studies on polyurethane (PU), argon (Ar) and Medpor scaffolds. **a** DNA quantification assay confirmed greater cell attachment to Ar scaffolds compared to Medpor and PU (Ar vs PU < 0.05, Ar vs Medpor *p < 0.05). **b** DNA quantification assay confirmed greater DNA content at 1, 4, 7 and 10 days on Ar scaffolds compared to Medpor and PU (Ar vs PU < 0.05, Ar vs Medpor p < 0.05). PU; Unmodified polyurethane

At 24 h macrophage adhesion was the greatest on Ar scaffolds compared to Medpor and PU scaffolds (Fig. 5a). There was greater adhesion of macrophages on Medpor surfaces compared to PU (p < 0.05) (Fig. 5a). From days 2–14 days the macrophage DNA content was significantly greater on Ar scaffolds compared to PU and Medpor (p < 0.05) (Fig. 5b) F-actin was used to study macrophage morphology. The actin staining demonstrated more elongated cells (red arrows) on the Medpor and Ar scaffolds than the PU scaffolds (Additional file 1: Figure S1). ELISA technique was used to study cytokines release by the macrophages on the different surfaces (Additional file 2: Figure S2). Macrophages on Medpor scaffolds showed enhanced secretion of IL-1β, and TNF-α cytokines compared to PU and Ar scaffolds at 4, 7 and 10 days. The macrophage secretion of IL-10 was the greatest on the Ar scaffolds at 7 and 10 days compared to PU and Medpor scaffolds (Additional file 2: Figure S2).

### In vivo assessment of tissue integration and angiogenesis

**Fig. 6:**
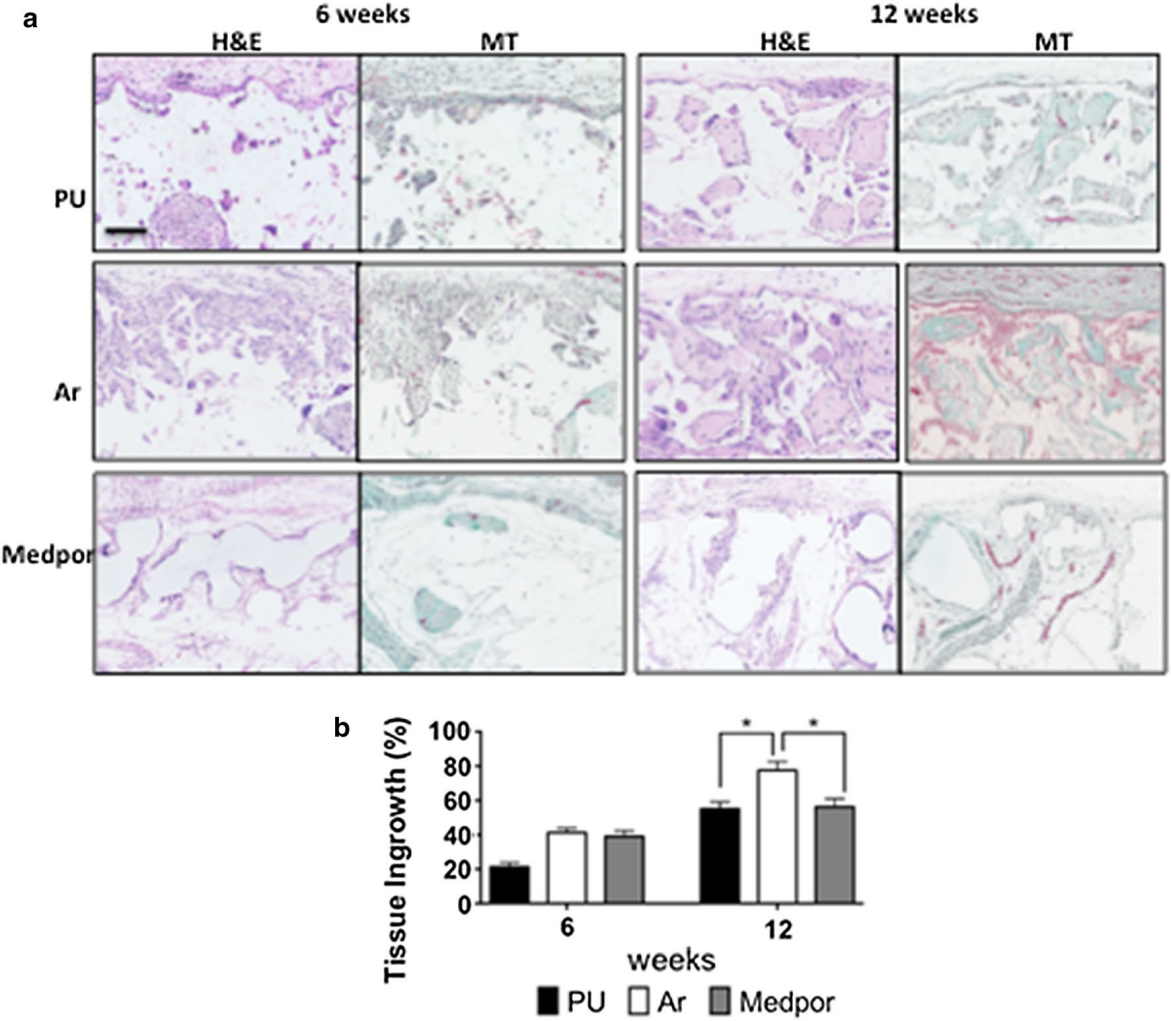
Analysis of tissue integration and angiogenesis of polyurethane (PU), argon (Ar) and Medpor scaffolds following over subcutaneous implantation in a mouse model at 12 weeks. **a** H&E analysis and Masson Trichrome staining of tissue ingrowth of the PU, Ar and Medpor scaffolds. **b** Quantification of the tissue ingrowth over 12 weeks. Scale bar refers to 250 μm. P values * < 0.05. PU; Unmodified polyurethane

**Fig. 7:**
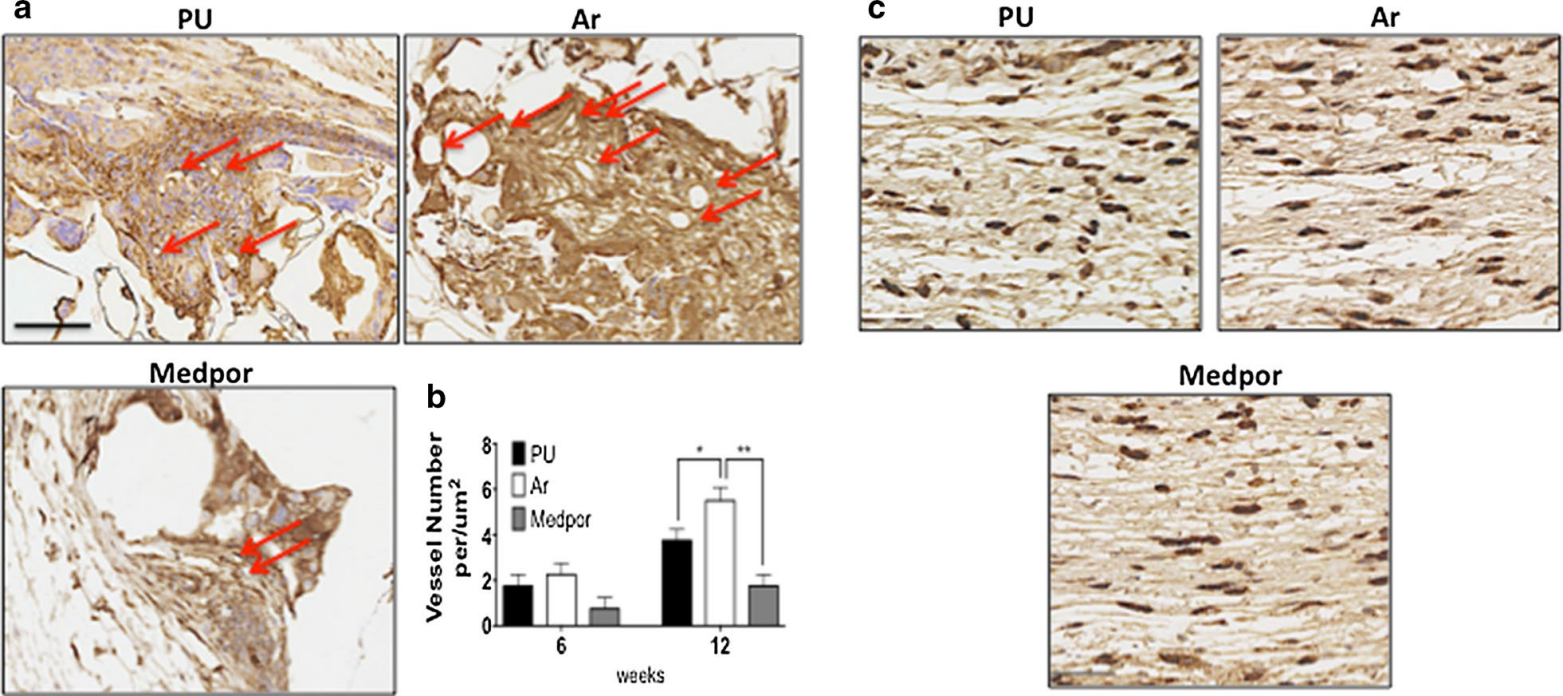
Angiogenesis and immune response analysis of the polyurethane (PU), argon (Ar) and Medpor scaffolds over 12 weeks in a subcutaneous implantation in a mouse model. **a** C31 staining after 12 weeks. **b** Quantification of vessel formation after 12 weeks. Scale bar refers to 250 μm. P values * < 0.05 ** < 0.01. **c** Macrophage infiltration over 12 weeks of the PU, Ar and Medpor scaffolds. CD68 staining was similar between the scaffolds within the pores. Scar bar refers to 50 μm. PU; Unmodified polyurethane

Scaffolds of PU, Ar, and Medpor were implanted subcutaneously for 12 weeks to assess tissue integration and angiogenesis. H&E staining was used to compare levels of tissue integration between the scaffolds (Fig. 6a, b). At 6 weeks Ar and Medpor showed similar levels of tissue integration, which was significantly higher than PU scaffolds (p < 0.05). The tissue ingrowth at 12 weeks of Ar scaffolds had doubled and significantly greater than the Medpor and PU scaffolds (p < 0.05). The PU and Medpor scaffolds showed similar tissue ingrowth at 12 weeks (Fig. 6). Massons trichrome demonstrated that Ar showed the greatest levels of integration by 12 weeks (p < 0.05). Masson Trichrome also demonstrated similar levels of integration of PU and Medpor scaffolds at 12 weeks (Fig. 6). To compare levels of angiogenesis CD31 staining was used to identify blood vessels within the newly formed tissue (Fig. 7). At 12 weeks, Ar showed greater CD31 staining than PU and Medpor (Fig. 7a, b). CD31 staining was also greater on the PU scaffolds than Medpor at 12 weeks (p < 0.05). CD68 stained the macrophages in the newly formed tissue to assess the immune response between the scaffolds. There were similar levels of CD68 positive cells on the three scaffolds after 12 weeks (Fig. 7c).

## Discussion

In this study, a nanocomposite polyurethane (PU) was compared with argon modified polyurethane and clinically available Medpor to determine its applicability to be used for facial cartilage reconstruction. The three material surfaces were compared for levels of tissue integration and angiogenesis. Argon polyurethane nanocomposite scaffolds supported cell attachment, proliferation and ECM formation in vitro as well as tissue integration and angiogenesis in vivo.

Protein adsorption is vital to determining cell adhesion onto biomaterials surfaces [33]. The Ar surfaces demonstrated the highest protein adsorption when compared to Medpor surfaces. The greater protein adsorption on the Ar scaffolds could have provided a greater number of anchorage sites for cell attachment. Protein conformation studies using fibronectin and vitronectin were also conducted to understand protein adsorption on the surfaces in more detail. The two proteins showed higher conformation on Ar scaffolds than on PU and Medpor surfaces allowing for greater bioactivity of the protein and consequently greater cell adhesion. The upregulation of cell adhesion related genes including vinculin, talin, and paxillin by the HDFs on the Ar compared to Medpor scaffolds, highlights the greater cell adhesion on the Ar surfaces. Cell growth is influenced by cell adhesion to the biomaterial surface. The Ar scaffolds allowed for greater cell adhesion due to enhanced protein adsorption compared to Medpor scaffolds, which led to greater growth over time in vitro.

The protein adsorption was varied on the scaffolds due to the differences in the surface properties of PU, Ar and Medpor scaffolds (Table 1). The three scaffolds had varied tensile mechanical properties. For example Medpor had a Young’s Elastic Modulus of 140.09 ± 0.04 in tension, which is 35-fold times higher than Ar scaffolds. The surface topography of the scaffolds was also different, with varying roughness values between the three surfaces. Ar scaffolds have a roughness of 12 ± 2 nm, whereas Medpor has a roughness of 190 nm using AFM analysis. The surface wettability is also different for example the water contact angle of Ar scaffolds is on average 12° compared to Medpor at 105°. In summary, Ar scaffolds are less stiff, less rough and less hydrophobic than Medpor scaffolds. Protein adsorption onto a material surface is influenced by surface roughness and surface hydrophobicity and can determine protein quantity and conformation [34]. The differences in the scaffold’s topography but not mechanical properties may have accounted for the varied tissue integration and vascularization response observed in this study.

**Table 1:** Comparison of polyurethane, argon modified polyurethane and Medpor scaffolds characteristics including surface roughness, contact angle and mechanical properties

Biomaterial characteristic (average)	PU	Medpor	Argon modified PU
Surface roughness (Rq)	8 ± 1	119.0 ± 13.8^a^	11 ± 2
Contact angle (Degrees)	67 ± 7	45.67° ± 0.23^a^	12 ± 2
Tension Young’s elastic modulus (MPa)	4.2 ± 0.04	140.09 ± 0.04^a^	4.2 ± 0.8

*PU* polyurethane

^a^Data is reproduced from Nayyer et al. Nanomedicine. 2014;10:235–46

Reports have demonstrated that protein adsorption is greater on hydrophobic surfaces [34–36]. However, there are also reports that have shown that protein adsorption can be varied on hydrophilic surfaces and protein adsorption can occur on hydrophilic surfaces [25]. Protein adsorption depends on the difference in the charge interaction and protein conformation of the scaffolds [25]. The function of a protein is determined by the protein conformation and the activity of the protein is controlled by the orientation and or its conformation [37]. The greater cell adhesion and growth observation observed on Ar scaffolds than Medpor could be due to both increased protein adsorption and more proteins were adsorbed in optimal conformations despite Ar scaffolds having a hydrophilic surface. With optimal proteins adsorption, more cells were able to adhere to the Ar scaffold to form tissue and allow for vascularisation in vivo.

Surface roughness is also important in determining protein adsorption to a scaffold surface [36]. Medpor was shown to have a rougher surface than Ar. Studies have shown that increasing the roughness of a surface may increase the surface area for potential proteins adsorption [38]. However, the rougher surface may have also prevented the proteins to be adsorbed in an optimal conformation and created inactive reactive sites. The smoother surface of Ar compared to Medpor may have provided an optimal surface topography for protein adsorption in the correct configuration to induce the desired cell responses. The greater protein adsorption on Ar scaffolds would have allowed for greater cell adhesion, which would have contributed to greater tissue formation and vascularisation in vivo. It is important to highlight that protein adsorption is a complex process and does not only depend on vitronectin and fibronectin adsorption, which were the proteins evaluated in this study. After implantation in vivo, several protein adsorb onto the implant surface including plasma proteins, albumin, fibrinogen and many more [39–41]. Hence, future studies must examine the behaviour of several other proteins in human plasma not only vitronectin and fibronectin to completely comprehend the protein-surface interaction that is occurring on the Ar scaffolds.

The subcutaneous implantation of the scaffolds allowed for assessment of tissue integration and angiogenesis over 12 weeks. Tissue integration and angiogenesis are vital to allow for anchorage of the implant in vivo [42]. The Ar scaffolds allowed for greater tissue formation at 12 weeks compared to Medpor. The upregulation of genes associated with the formation of extracellular matrix including collagen type I, elastin and laminin by the HDFs on the Ar scaffolds may have allowed for a greater tissue ingrowth in vivo. The Masson Trichrome staining also highlighted the greater collagen formation in vivo on the Ar scaffolds. The greater cell adhesion on the Ar scaffolds due to improved protein adsorption allowed for greater cell growth, which allowed for greater ECM production in vitro and in vivo.

Vascularisation of the scaffolds in vivo was determined by CD31 staining. At 6 weeks all scaffolds shown minimal vessel formation but by 12 weeks Ar scaffolds allowed for greater vessel ingrowth compared to Medpor. The VEGF growth factor is known to induce pathways responsible for vessel formation [43]. The HDFs on Ar scaffolds demonstrated increased levels of VEGF secretion as well as mRNA expression of VEGF in vitro compared to Medpor, which may have allowed for greater vascularisation in vivo.

The immune response of the scaffolds was examined in vitro and in vivo. The greater macrophage adhesion onto the Ar scaffolds could be due to the greater protein adsorption observed on the scaffolds. The greater macrophage adhesion will have allowed for greater DNA content in culture over time. The release of the inflammatory cytokines mediators was important to examine on the different scaffolds to understand the inflammatory potential of the scaffolds. The pro-inflammatory mediators IL-1β and TNF-α, are known to be secreted by activated macrophage to regulate inflammation [44]. The IL-1β cytokine, is secreted by activated macrophages and governs inflammatory responses by modifying cell proliferation, differentiation and apoptosis [45]. The IL-10 cytokine, is considered an anti-inflammatory cytokine, secreted by activated macrophages and promotes healing and reduces inflammation [46]. In this study, macrophages on Ar and PU scaffolds showed similar levels of cytokine mediators. On the other hand the macrophages on the Medpor showed greater levels of pro-inflammatory mediators and lower levels of anti-inflammatory mediator IL-10 than PU and Ar.

All biomaterials undergo a foreign body response to the host tissue when implanted subcutaneously [47]. It is a structured sequence of events to allow for the host tissue to accept the implant. To determine if the observed differences in the secretion of cytokines and behaviour of the macrophages caused any difference to the foreign body response the immune response to the scaffolds was examined in vivo. The CD68 macrophages were examined in vivo over 12 week on all three scaffolds. There was a similar decrease in the macrophage response over the 12 weeks. Typically all implants trigger the attachment and recruitment of inflammatory cells as apart of the foreign body response reaction when a biomaterial is implanted [47]. Macrophages not only govern the inflammatory response by secreting chemokine, cytokines and growth factors but are also important in tissue regeneration [48]. Despite observing differences in the in vitro macrophage responses, no differences were observed in the in vivo recruitment of macrophages to alter the immune response of the three scaffolds. Future work, will examine the proportion of activated and polarised macrophages and their M1 or M2 phenotype to understand the significance of the in vitro findings and to understand their role in modification of inflammation and regeneration. The M1 phenotype is involved in the killing of pathogens as a pro-inflammatory response [49–51] where as the M2 macrophages suppress the intracellular killing processes and reduction of pro-inflammatory responses by producing IL-10 [52]. The ELISA analysis may indicate there is greater amount of M2 macrophages on the Ar scaffolds due to the higher levels of IL-10 secretion.

Surface modification of scaffolds aims to provide appropriate responses to induce desired cell responses. This study has shown that PSM using argon gas provides surface topographical and chemical signals to allow for greater tissue formation and vascularisation without influencing the scaffold properties or cell biocompatibility. Argon improved tissue formation and angiogenesis by creating an optimal surface for protein adsorption and orientation to allow for desired cell adhesion and growth. PSM can be easily used to modify other biomaterials including natural and synthetic scaffolds without altering their structural properties. Argon modification overcomes the limitations with current physical and chemical surface modification techniques including problems with protein half-life and stability in vivo. This study provides a simple tool by which scaffolds can be modified to improve tissue integration and angiogenesis.

## Conclusions

This study has provided extensive data that Ar nanocomposites scaffolds allowed for cell adhesion, growth and in vivo tissue ingrowth and vascularisation to a greater degree than clinically used Medpor. Argon modified polyurethane scaffolds have demonstrated to be a promising biomaterial to replace missing or damaged auricular cartilage. This study provides evidence that nanocomposites polyurethanes are promising to replace cartilage and can be explored further as a biomaterial for cartilage tissue engineering applications.

## Additional files

**Additional file 1: Figure S1.** Human macrophage morphology on polyurethane (PU), argon (Ar) and Medpor scaffolds after 24 h. F-actin staining demonstrates more elongated cells (red arrow) on the Medpor and Ar scaffolds than the PU scaffold. Some macrophages showed evidence of pseudopodia (yellow arrow). Scale bar refers to 50 μm. PU; Unmodified Polyurethane.

**Additional file 2: Figure S2.** Cytokine profile secreted from the macrophages after 10 days in vitro on polyurethane (PU), argon (Ar) and Medpor scaffolds. [A] Tumor necrosis factor-α (TNF-α) secretion was significantly greater on Medpor than PU and Ar scaffolds at 4, 7 and 10 days (*p < 0.05). [B] Interleukin-1β (IL-β) secretion was significantly greater on Medpor than PU and Ar scaffolds at 2, 7 and 10 days (*p < 0.05). [C] Interleukin-10 (IL-10) secretion was significantly greater on Ar than PU and Medpor scaffolds at 4, 7 and 10 days (*p < 0.05). PU; Unmodified Polyurethane.
