# Supplementary figures and images for: Argon plasma modified nanocomposite polyurethane scaffolds provide an alternative strategy for cartilage tissue engineering
Source: J Nanobiotechnology. 2019 Apr 6;17:51. doi: 10.1186/s12951-019-0477-z (PMC6451776; doi:10.1186/s12951-019-0477-z)

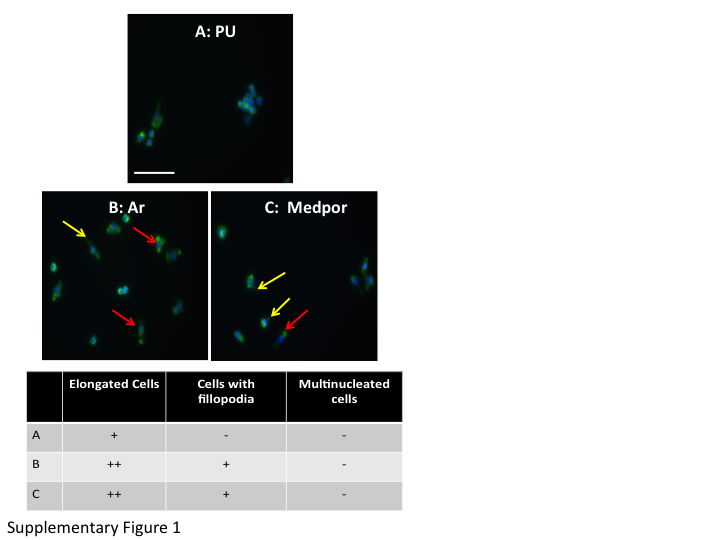

Supplement: Supplementary file 1 — Additional file 1: Figure S1. Human macrophage morphology on polyurethane (PU), argon (Ar) and Medpor scaffolds after 24 h. F-actin staining demonstrates more elongated cells (red arrow) on the Medpor and Ar scaffolds than the PU scaffold. Some macrophages showed evidence of pseudopodia (yellow arrow). Scale bar refers to 50 μm. PU; Unmodified Polyurethane. [file 12951_2019_477_MOESM1_ESM.tiff]

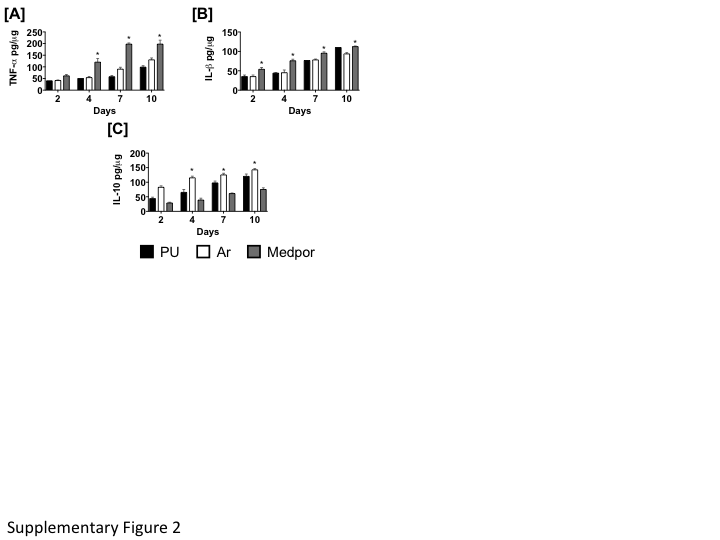

Supplement: Supplementary file 2 — Additional file 2: Figure S2. Cytokine profile secreted from the macrophages after 10 days in vitro on polyurethane (PU), argon (Ar) and Medpor scaffolds. [A] Tumor necrosis factor-α (TNF-α) secretion was significantly greater on Medpor than PU and Ar scaffolds at 4, 7 and 10 days (*p < 0.05). [B] Interleukin-1β (IL-β) secretion was significantly greater on Medpor than PU and Ar scaffolds at 2, 7 and 10 days (*p < 0.05). [C] Interleukin-10 (IL-10) secretion was significantly greater on Ar than PU and Medpor scaffolds at 4, 7 and 10 days (*p < 0.05). PU; Unmodified Polyurethane. [file 12951_2019_477_MOESM2_ESM.tiff]
